# Genome-Wide Association Analysis and Genetic Parameters for Egg Production Traits in Peking Ducks

**DOI:** 10.3390/ani14131891

**Published:** 2024-06-27

**Authors:** Jun Zhou, Jiang-Zhou Yu, Mei-Yi Zhu, Fang-Xi Yang, Jin-Ping Hao, Yong He, Xiao-Liang Zhu, Zhuo-Cheng Hou, Feng Zhu

**Affiliations:** 1 National Engineering Laboratory for Animal Breeding, Key Laboratory of Animal Genetics, Breeding and Reproduction of the Ministry of Agriculture, College of Animal Science and Technology, China Agricultural University, Beijing 100193, China; zhoujun138@cau.edu.cn (J.Z.); jzyu@cau.edu.cn (J.-Z.Y.); zmy_0430@163.com (M.-Y.Z.); 2 Beijing Nankou Duck Breeding Technology Co., Ltd., Beijing 102202, China; yangfangxi@163.com (F.-X.Y.); haojp73@126.com (J.-P.H.); 3 Cherry Valley Breeding Technology Co., Ltd., Beijing 100088, China; heyong_2008@126.com (Y.H.); zhuxl8769@163.com (X.-L.Z.)

**Keywords:** Peking ducks, egg production traits, genome-wide association study

## Abstract

**Simple Summary:**

Egg production is a priority for the poultry industry, particularly for traits such as the age at first egg, number of eggs at different stages, and laying rate. Although ducks have higher egg production capabilities than other poultry, the genetic factors influencing these traits have been minimally studied. In this study, we assessed the heritability of these traits and conducted a genome-wide association study, identifying nine significant genetic markers associated with the age at first egg and egg number. The results of the analyses suggest that the candidate variants may have an effect on egg production by influencing ovarian function and oocyte maturation processes. These findings contribute substantially to understanding the genetic foundations of egg production in ducks and provide a basis for future genetic advances in poultry.

**Abstract:**

Egg production traits are crucial in the poultry industry, including age at first egg (AFE), egg number (EN) at different stages, and laying rate (LR). Ducks exhibit higher egg production capacity than other poultry species, but the genetic mechanisms are still poorly understood. In this study, we collected egg-laying data of 618 Peking ducks from 22 to 66 weeks of age and genotyped them by whole-genome resequencing. Genetic parameters were calculated based on SNPs, and a genome-wide association study (GWAS) was performed for these traits. The SNP-based heritability of egg production traits ranged from 0.09 to 0.54. The GWAS identified nine significant SNP loci associated with AFE and egg number from 22 to 66 weeks. These loci showed that the corresponding alleles were positively correlated with a decrease in the traits. Moreover, three potential candidate genes (ENSAPLG00020011445, ENSAPLG00020012564, TMEM260) were identified. Functional enrichment analyses suggest that specific immune responses may have a critical impact on egg production capacity by influencing ovarian function and oocyte maturation processes. In conclusion, this study deepens the understanding of egg-laying genetics in Peking duck and provides a sound theoretical basis for future genetic improvement and genomic selection strategies in poultry.

## 1. Introduction

Laying traits have always been at the heart of research and improvement in the poultry industry. Eggs are one of the main sources of protein for consumers worldwide. Consequently, the egg production capacity of poultry has a direct impact on the food supply chain and the stability of the agricultural economy [1]. Egg number (EN) and laying rate (LR) serve as important phenotypic traits describing poultry egg production, which are not only related to the economic benefits of poultry farming, but also serve as key indicators for evaluating the improvement in outcomes of poultry breeds [2,3]. Similarly, age at first egg (AFE), as a key indicator of hen maturity, directly affects the egg-laying cycle of poultry and is an important measure of egg production performance [4]. In recent decades, systematic selection and breeding strategies have significantly improved the production and reproductive performance of laying hens [5]. Despite evident progress from traditional breeding methods, the complex genetic basis of egg production traits remains a significant research challenge for further genetic improvements.

In contrast to other poultry, such as chickens, domestic ducks exhibit unique physiological and reproductive attributes. For instance, they possess a relatively shorter egg-laying cycle, and their hormonal regulation mechanisms differ [6]. These factors directly influence the production rate and quality of their eggs. In recent years, with the rapid development of high-throughput sequencing technology and other biotechnologies, the reduction in cost has enabled the application of GWAS in a wider range of poultry genetic studies, successfully identifying a number of genetic markers associated with egg-laying traits [3,4,7,8,9,10]. However, there are still few studies related to egg-laying traits in ducks compared to chickens [2,11,12]. The genetic mechanism behind this high egg production capacity in ducks remains unclear.

Therefore, in this study, genetic parameter estimation and GWAS analyses of Peking duck laying traits (AFE, EN, LR) were performed to determine the inheritance patterns of Peking duck laying traits as well as trait-associated SNPs and candidate genes. By comparing the data with other poultry breed studies, we aspire to elucidate the genetic characteristics of the Peking duck in terms of laying traits further. This will provide robust theoretical backing and practical guidelines for future genetic improvements in poultry.

## 2. Materials and Methods

### 2.1. Population and Trait Measurements

In this study, 630 purebred Peking ducks were used. Animals are from the same strain and hatched in the same batch. The age at first egg (AFE) was recorded for each individual. Their egg production was recorded daily from the time they first laid eggs until 66 weeks of age. Analyzing the egg production curve (Figure S1), we segmented their laying cycle into three phases: the pre-peak phase (22–30 weeks, EN1), the peak phase (31–55 weeks, EN2), and the persistent phase (56–66 weeks, EN3). Cumulative egg production was then determined for each duck: from 22–36 weeks (EN22-36WK), up to 51 weeks (EN22-51WK), and culminating at 66 weeks (EN22-66WK). We also determined the laying rate (LR) as the percentage of the total eggs laid to the number of laying days between 28 and 56 weeks. Throughout this study, a standardized diet was maintained, and the ducks were nurtured under identical conditions.

### 2.2. Phenotypic Correction and Genetic Parameter Estimation

Prior to data analysis, all phenotypic data were subjected to rigorous screening, with only values within the range of [mean ± 3 standard deviations (SDs)] being retained [3,13]. Shapiro–Wilk tests were employed to verify the normality of all phenotypes [14]. Any data that deviated from the normal distribution were rescaled using a rank-based inverse transformation to match subsequent analyses [15].

The genetic parameters for egg production traits based on SNP were estimated using the linear mixed model incorporated in the ASReml-R (v4.1.0.176) package, articulated as

*y* = 1*μ* + *Zu* + *e*, (1)

In this framework, *y* epitomizes the phenotypic value of the trait; 1 and *Z* serve as the design matrices tied to the fixed effects (population mean) and the random effects (individual additive genetic effects), respectively; *µ* and *u* are the vectors designated for the fixed and random additive effects, respectively; and *e* signifies the random residual effects. 

### 2.3. Genotyping and SNP Identification

Fresh blood samples were obtained from the metatarsal vein using standard venipuncture methods and vacuum blood collection tubes. Subsequently, genomic DNA was successfully extracted from the obtained blood samples by using a DNA extraction kit (QIAampR DNA Blood Mini Kit; QIAGEN). This DNA sample underwent whole-genome resequencing on the DNBSEQ-T7 platform, adopting a 150 bp paired-end read strategy, with an average sequencing depth of 2.06×. For further analysis, the obtained reads were mapped to the mallard duck’s reference genome ASM874695v1 [16] using BWA(v0.7.10) software [17]. After alignment, SNP calling was carried out using the GATK haplotypecaller (v4.1) [18], where the main parameter settings were kept default, only modifying -stand_call_conf to 30. Subsequently, genotype imputation was performed using STITCH (v1.6.10) [19]. To ensure data quality, we employed quality control using VCFtools (--min-alleles 2, --max-alleles 2) (v0.1.16) [20] and PLINK (--geno 0.05 --maf 0.01 --mind 0.05) (v 1.90) [21]. After quality control, 581 individuals and 1,111,649 SNPs were kept for GWAS analysis.

### 2.4. GWAS Analysis

In order to obtain an independent locus, we leveraged the --indep-pairwise 50 5 0.2 parameter in PLINK, extracting autonomous SNPs. Then, principal component analysis (PCA) was performed, harnessing the isolated 42,623 markers. Addressing potential distortions from population stratification (Figure S2), significant principal components (*p* < 0.05) were integrated as covariates in a GWAS assessment [22]. We carried out the GWAS using the mixed-model approach based on GEMMA (v 0.98) software [23]. For every discerned trait, the association scrutiny was anchored on a univariate linear-mixed-model methodology. The ensuing statistical archetype is expressed as

*y* = *Wα* + *xβ* + *u* + *ε*, (2)

In this model, the vector *y* represents the trait values of all individuals; *W* is the covariate matrix containing fixed effects, which include significant principal components (*p* < 0.05) and a column of 1 s; *α* is the vector of corresponding coefficients, including the intercept; *x* is the vector of marker genotypes; *β* represents the effect size of the markers; *u* is the vector of individual random effects; and *ε* is the error vector. Wald test statistics are employed as the criterion for screening SNPs significantly associated with the trait under study. To determine the genome-wide significance and suggestive significance thresholds, considering that the Bonferroni correction is stringent [24], independent loci were pinpointed using the PLINK directive --indep-pairwise 50 5 0.2, rendering them as N (42,623) to ascertain the threshold. Consequently, genome-wide significance and suggestive significance thresholds were derived as 1.17 × 10^−6^ (0.05/42,623) and 2.35 × 10^−5^ (1.00/42,623), respectively. Illustrative Manhattan and QQ plots for each discerned trait were formulated employing the CMplot (v4.3.1) toolkit within the R programming environment [25].

### 2.5. Post-GWAS Exploration

To assess the association between specific loci and unique traits, as well as their impact on phenotypic variation, the phenotypic variance explained (PVE) for each significant or suggestive significant SNP locus was calculated [26]. LDBlockShow [27] was used to analyze linkage disequilibrium (LD) in genomic intervals. In this analysis, regions with D’ values exceeding 0.8 were defined as a block, where D’ is a measure for assessing LD. Then, to reduce the occurrence of false positives, conditional analyses were performed using the most significant SNP as a covariate [28,29]. We extracted genotypes of SNPs that were significant at the genome-wide level for different traits and, in conjunction with phenotypic traits, used non-parametric tests (Mann–Whitney U Test and Kruskal–Wallis Test) to statistically analyze whether significant phenotypic variation existed between different genotypes. To conduct detailed gene annotation, SnpEff (v5.0.1) [30] were used. Subsequently, functional enrichment analysis of the annotated candidate genes was performed using Metascape [31].

## 3. Results and Discussion

### 3.1. Phenotype and Genetic Parameter 

In this research, we performed a detailed analysis and descriptive statistics on the egg production traits of 618 Peking ducks (Table 1). The findings revealed that ducks in this population, on average, laid their first egg at the age of 172.52 days, with the AFE ranging between 146 and 203 days. Additionally, the average cumulative egg production of this population was 247.64 eggs from 22 to 66 weeks of age, demonstrating their stable egg-laying performance. Notably, during 28 to 56 weeks of age, individuals with excellent egg-laying performance could achieve a laying rate of 100%. The number of eggs laid by these individuals was sometimes recorded as two on certain days. These two eggs might be calcified simultaneously in the uterus or formed within a short egg formation cycle [32]. This provides a solid genetic foundation for further enhancing the egg production traits of the population. The average AFE of Shaoxing ducks was 136.95 days, which is earlier compared to the Peking duck population, and their average egg production from the start of laying to 66 weeks of age was 268.96, which is more prominent than that of the Peking duck population [11]. This is mainly due to the fact that Shaoxing ducks are mainly egg-laying breeds with a long history of selection for egg-laying traits, whereas Peking ducks are mainly meat-laying breeds. This further suggests that there is still considerable room for improvement in the egg-laying performance of Peking ducks.

**Figure 1:**
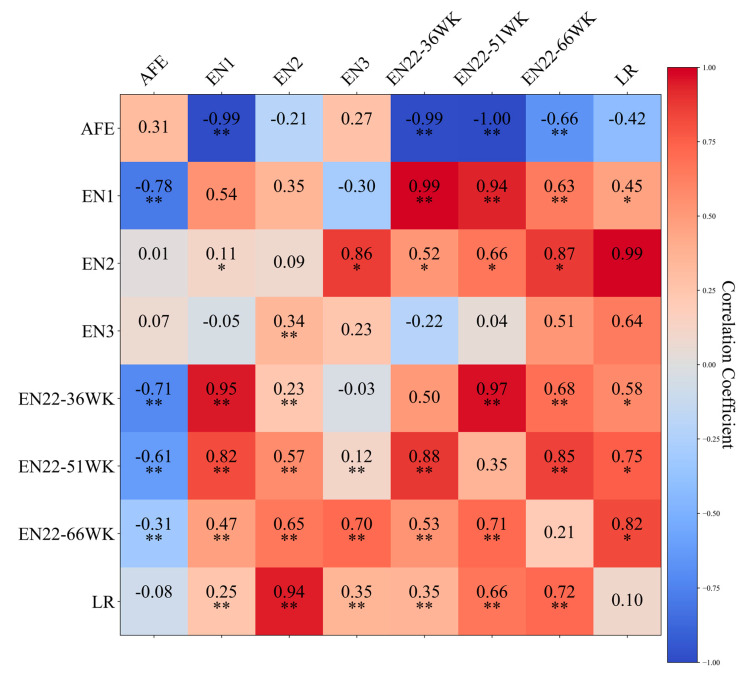
Genetic parameters of age at first egg, egg number, and egg laying rate based on SNP information. The diagonal displays the estimated results of heritability for each trait, the upper triangle shows the estimated results of genetic correlation coefficients between each trait, and the lower triangle shows the estimated results of phenotypic correlation coefficients between each trait. In the correlation coefficients between two traits, “**” indicates *p* < 0.01, and “*” signifies 0.01 < *p* < 0.05. AFE, age at first egg; EN1, egg number in the pre-peak laying period from 22 to 30 weeks of age; EN2, egg number in the peak laying period from 31 to 55 weeks of age; EN3, egg number in the persistent laying period from 56 to 66 weeks of age; EN22-36WK, egg number from 22 to 36 weeks of age; EN22-51WK, egg number from 22 to 51 weeks of age; EN22-66WK, egg number from 22 to 66 weeks of age; LR, egg laying rate from 28 to 56 weeks of age.

The heritability, genetic correlation coefficients, and phenotypic correlation coefficients for each phenotypic trait, based on SNP information, are all depicted in Figure 1. The results of this study uncover that before reaching the peak of the weekly laying rate (36 weeks of age, Figure S1), the heritability of the number of eggs laid is approximately 0.5, indicating a high level. Concurrently, the heritability of the EN2 is only 0.09, and the LR from 28 to 56 weeks of age is 0.10, both of which are classified as low heritability traits. Our results are similar to those of other studies. Yuan et al. used the egg production records of 1534 F2 generation hens to estimate genetic parameters and conducted a GWAS, finding heritability ranging between 0.17 and 0.36 for traits such as EN, LR, and AFE [3]. Xu et al. performed a GWAS and haplotype-sharing analysis for Shaoxing ducks, revealing heritability for AFE, egg numbers at 43 weeks, and 66 weeks of age as 0.15, 0.20, and 0.22, respectively [11]. Notably, in the three cumulative egg-laying cycles, as time progresses, the heritability of each trait demonstrates a gradually declining trend (0.50, 0.35, 0.21). This trend reveals that as time extends, the influence of genetic factors on the egg-laying performance of Peking ducks decreases. This has significant guiding significance for the production management and genetic improvement in Peking ducks. In addition, the wide range of heritability measurements indicates that genetic and environmental factors may have different weights at different growth stages and conditions. This has important implications for the design of genetic improvement strategies and precision management. 

### 3.2. GWA Analysis

**Figure 2:**
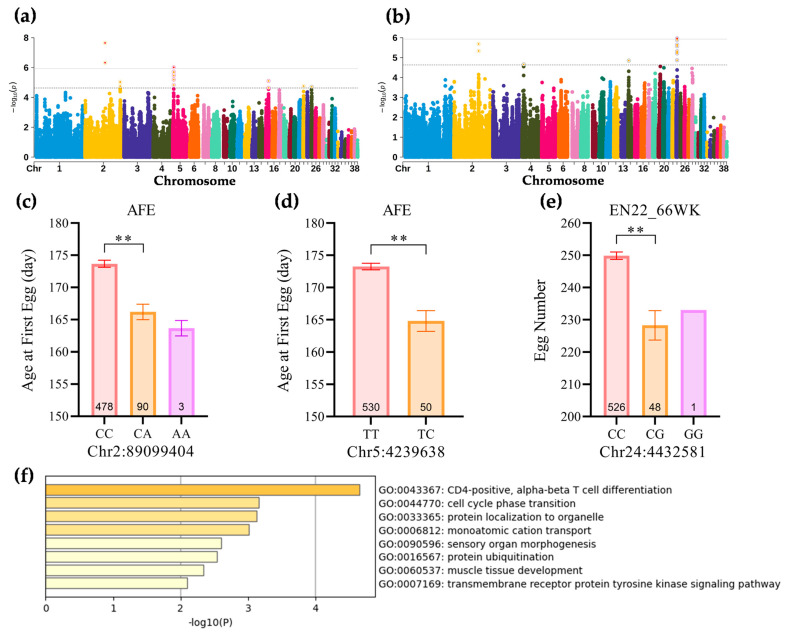
Manhattan plot of genome-wide association study for egg production traits; statistical comparison of genotype and phenotype for the most significant SNPs, and gene functional enrichment analysis. (**a**): AFE, age at first egg. (**b**): EN22-66WK, egg number from 22 to 66 weeks of age. Each dot represents an SNP in the dataset. The horizontal gray line and gray dashed line indicate the genome-wise significance threshold (*p*-value = 1.17 × 10^−6^) and genome-wise suggestive significance threshold (*p*-value = 2.35 × 10−5), respectively. (**c**): AFE and three genotypes of the SNP Chr2:89099404 (C/A). (**d**): AFE and two genotypes of the SNP Chr5:4239638 (T/C). (**e**): EN22-66WK and three genotypes of the SNP Chr24:4432581 (C/G). “**” indicates that there is a significant phenotypic difference between different genotypes according to the non-parametric test (*p* < 0.01). (**f**): Functional enrichment analysis was conducted on the candidate genes annotated from significant SNPs in different egg production traits.

We successfully identified 248 SNPs significantly associated with these egg production traits (Table 2 and Table S1; Figure 2, Figures S3 and S4). Only in the two traits, AFE and EN22-66WK, SNPs that reached the genome-wide significance level were detected. The characteristics of the markers significantly related to AFE and EN22-66WK are summarized in Table 2. For the AFE, four significantly associated SNPs were detected on chromosomes 2 and 5 (Table 2; Figure 2, Figures S4a and S5). Among them, the most significant SNP was located at 89,099,404 bp on chromosome 2 (−log(*p*) = 7.65, PVE = 3.39%). Additionally, 66 suggestively associated SNPs were located at specific positions on chromosomes 2, 5, 15, 23, and 25 (Table S1). For EN22-66WK): five significantly associated SNPs were observed, all located on chromosome 24 (Table 2; Figure 2, Figures S4g and S5). The most significant SNPs were at 4,432,581 bp and 4,432,657 bp (−log(*p*) = 5.97, PVE = 2.51%), with other significant SNPs at 4,432,015 bp, 4,432,044 bp, and 4,432,523 bp. Moreover, 27 SNPs suggestively associated with EN22-66WK were found on chromosomes 2, 4, 14, and 24. For LR (Table S1; Figures S3f and S4h), only one suggestively associated SNP was observed, located at 109,888,590 bp on chromosome 2. Additionally, on chromosomes 2, 5, 17, and 28, a total of 20 SNPs were associated with multiple egg production traits (AFE, EN) (Table S2).

After annotation, 41 candidate genes were identified (Tables S3 and S4). The functional enrichment analysis revealed multiple significant biological processes related to the egg-laying traits of Peking ducks (Figure 2f; Table S5). Among them, the most significant GO Term is CD4-positive; alpha–beta T cell differentiation related to T cell differentiation (GO:0043367, −log(*p*) = 4.65). In this process, seven genes potentially associated with egg-laying traits were identified, namely STAT3, CD83, FOXP1, JARID2, PPP3CA, UBASH3B, and KIF23. In previous studies, Yuan et al. [3] reported that the egg-laying traits of laying hens are associated with immunity and cytokines, which play a critical regulatory role in the modulation of ovarian function [33]. Additionally, the cell cycle phase transition associated with cell cycle transitions (GO:0044770, −log(*p*) = 3.16) was also identified as a significant biological process related to the egg-laying traits of Peking ducks. The regulation of the cell cycle is closely related to the formation, maturation, and subsequent release of oocytes [34,35], further emphasizing its importance in regulating the egg-laying traits of Peking ducks. Moreover, the “protein ubiquitination” process involves protein stability and degradation (GO:0016567), which is biologically crucial for ensuring the normal development and maturation of oocytes [36]. Meanwhile, the results showed a significant enrichment of the “transmembrane receptor protein tyrosine kinase signaling pathway” (GO:0007169), suggesting that this signaling pathway might play a key role in regulating the development and maturation of oocytes [37]. Among the SNPs that are significant at the whole-genome level, three candidate genes were found (Table 2), including TMEM260. In previous studies, mutations in the TMEM260 gene have been proven to be associated with neurodevelopment, cardiac, and renal syndrome in children [38]. This study also revealed that the long isoform of TMEM260 is enriched on the cell membrane, which may be closely related to the pathogenesis of the aforementioned diseases [38]. However, in the field of poultry, especially in the study of egg-laying traits, the function of the TMEM260 gene is not fully clear. Further experiments and studies are necessary to clarify the specific role and potential molecular mechanisms of TMEM260 in poultry egg-laying traits.

The accuracy of genome-wide association analysis and genetic parameter estimation is generally affected by sample size. In this study, a total of 618 ducks were used for analysis. The results showed that the sample size of this study could meet the power requirements of the analyses. Of course, the larger the sample size, the higher the sensitivity of QTL mapping. This is especially true for some multi-gene regulated phenotypes. For example, although both EN1 and EN22-36WK are traits of moderate heritability, no significant loci were detected at the genome level. This is most likely due to the fact that the haplotypes causing variation in these traits have a small effect on the phenotype. Increasing the effective sample size in future studies may lead to better detection of these QTL.

## 4. Conclusions

In this study, leveraging individual genotype data, we conducted a genome-wide association analysis on the egg production traits of the Peking duck. Moreover, we calculated genetic parameters based on SNPs, and the results revealed that the heritability estimates for various egg production traits ranged from 0.09 to 0.54. Notably, there was a declining trend in the heritability estimates for cumulative egg number over time, decreasing from 0.50 to 0.35, and further to 0.21. Through rigorous screening, we identified a total of 248 SNPs significantly associated with these traits. Among them, nine SNPs reached the genome-wide significance level, while the rest were potentially significant SNP sites. Through gene annotation analysis, we pinpointed 41 potential candidate genes associated with egg production traits, notably, 11 of which are newly reported genes, which were involved in the regulation of the cell cycle, oocyte formation, maturation, and release, as well as certain specific immune responses; all these genes profoundly influence the egg production characteristics of the Peking duck. These novel findings not only enrich our understanding of the genetic and physiological mechanisms in poultry but also provide practical guidance and invaluable scientific evidence for the genetic improvement in poultry.

## Figures and Tables

**Table 1:** Descriptive statistics of egg production traits for Peking ducks.

Traits ^1^	Num ^2^	Mean	SD ^3^	CV ^4^ (%)	Min	Max
AFE (day)	618	172.52	12.05	6.98	146	203
EN1	618	31.47	11.67	37.09	3	63
EN2	602	161.86	11.22	6.93	117	175
EN3	614	55.31	17.65	31.91	1	77
EN22-36WK	615	70.52	12.71	18.02	34	104
EN22-51WK	607	168.20	15.37	9.14	115	206
EN22-66WK	612	247.64	27.89	11.26	159	299
LR (%)	604	91.56	6.58	7.18	67	100

Note: ^1^: AFE, age at first egg; EN1, egg number in the pre-peak laying period from 22 to 30 weeks of age; EN2, egg number in the peak laying period from 31 to 55 weeks of age; EN3, egg number in the persistent laying period from 56 to 66 weeks of age; EN22-36WK, egg number from 22 to 36 weeks of age; EN22-51WK, egg number from 22 to 51 weeks of age; EN22-66WK, egg number from 22 to 66 weeks of age; LR, egg laying rate from 28 to 56 weeks of age. ^2^: Number of ducks that pass quality control of phenotypic value. ^3^: Standard deviation. ^4^: Coefficient of variation.

**Table 2:** The information on SNPs that show significant associations with the age at first egg (AFE) and the number of eggs produced from 22 to 66 weeks of age (EN22-66WK) at the whole-genome level (*p* < 1.17 × 10^−6^).

Traits ^1^	Chr	Position	Maf ^2^	Beta ± SE ^3^	−log(*p*)	PVE (%) ^4^	Candidate/Nearest Gene	Location (kb) ^5^
AFE	2	89099404	0.084	−0.62 ± 0.11	7.65	3.39	ENSAPLG00020011445	D 38.701
	2	89104958	0.075	−0.59 ± 0.12	6.32	2.71	ENSAPLG00020011445	D 44.255
	5	4239638	0.043	−0.76 ± 0.15	6.01	2.38	TMEM260	Intron
	5	4261786	0.043	−0.76 ± 0.15	6.01	2.53	TMEM260	D 15.339
EN22-66WK	24	4432581	0.043	−0.73 ± 0.15	5.97	2.51	ENSAPLG00020012564	D 1.423
	24	4432657	0.043	−0.73 ± 0.15	5.97	2.51	ENSAPLG00020012564	D 1.347
	24	4432015	0.044	−0.73 ± 0.15	5.96	2.51	ENSAPLG00020012564	D 1.989
	24	4432044	0.044	−0.73 ± 0.15	5.96	2.51	ENSAPLG00020012564	D 1.96
	24	4432523	0.044	−0.73 ± 0.15	5.96	2.51	ENSAPLG00020012564	D 1.481

Note: ^1^: AFE, age at first egg; EN22-66WK, egg number from 22 to 66 weeks of age. ^2^: Maf, minor allele frequency. ^3^: Beta ± SE, regression coefficient plus or minus its standard error. ^4^: PVE, phenotypic variance explained. ^5^: D or U indicate that the SNP is upstream or downstream of the gene, respectively.

## Data Availability

The data used in this study was deposited at the figshare repository (https://doi.org/10.6084/m9.figshare.25001777.v1) (accessed on 16 January 2024).

## Supplementary Materials

The following supporting information can be downloaded at: https://www.mdpi.com/article/10.3390/ani14131891/s1. Table S1: Information for all significant SNPs related to egg production traits; Table S2: SNP loci that are significant across multiple egg production traits; Table S3: The number of significant SNP loci for different egg production traits on various chromosomes and the annotated candidate genes; Table S4: Annotation for candidate genes; Table S5: The biological pathway of candidate genes; Figure S1: Egg production curve of the Peking duck population in the laying cycle from 22 to 66 weeks of age. Each orange dot represents the laying rate in the respective week; Figure S2: Principal component analysis of population structure; Figure S3: Manhattan plot of genome-wide association study for egg production traits; Figure S4: QQ plot of genome-wide association study for egg production traits; Figure S5: Manhattan plot of association analysis for age at first egg and egg number from 22 to 66 weeks of age in specific intervals, linkage disequilibrium analysis, and conditional analysis.
