# Supplementary material for: Genome-Wide Association Analysis and Genetic Parameters for Egg Production Traits in Peking Ducks
Source: Animals (Basel). 2024 Jun 27;14(13):1891. doi: 10.3390/ani14131891 (PMC11240742; doi:10.3390/ani14131891)
Supplement: Supplementary file 1 [file animals-14-01891-s001.zip › supplementary_figure.pdf]

Supplementary Material

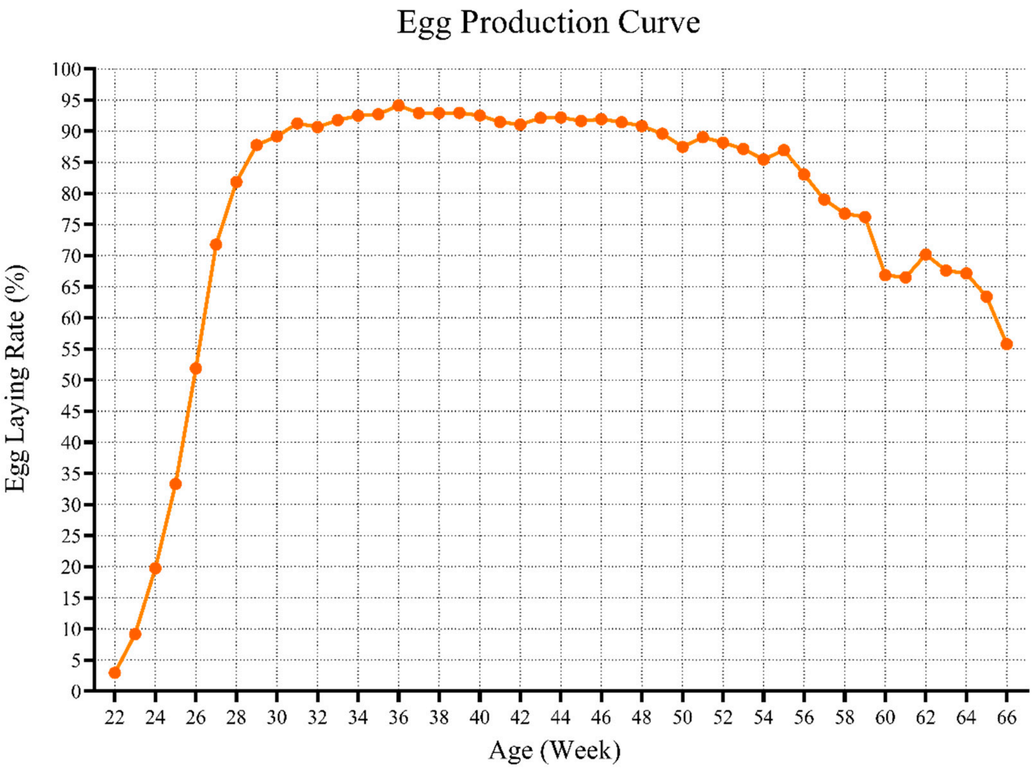

Figure S1. Egg production curve of the Peking duck population in the laying cycle from 22 to 66 weeks of age. Each orange dot represents the laying rate in the respective week.

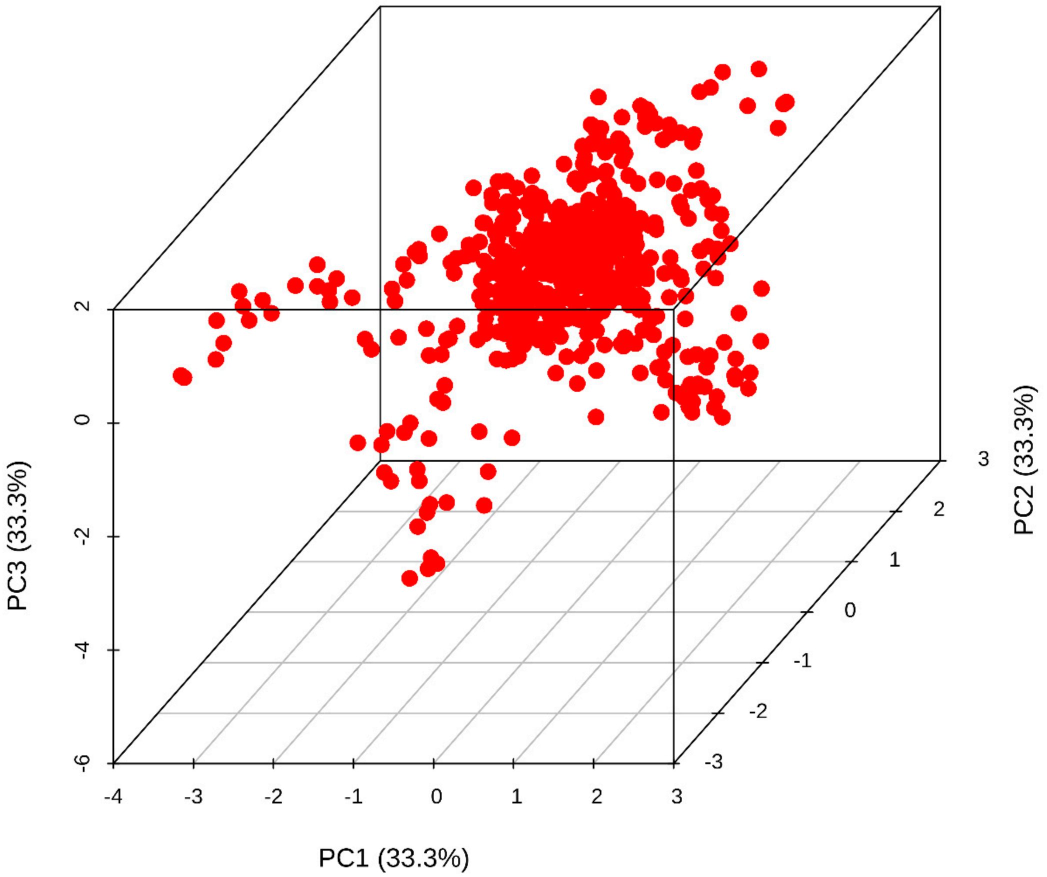

Figure S2. Principal component analysis of population structure.
Note: The results of the principal component analysis show that the first principal component (PC1) is not significant, indicating no obvious stratification within the population. Each dot represents an individual.

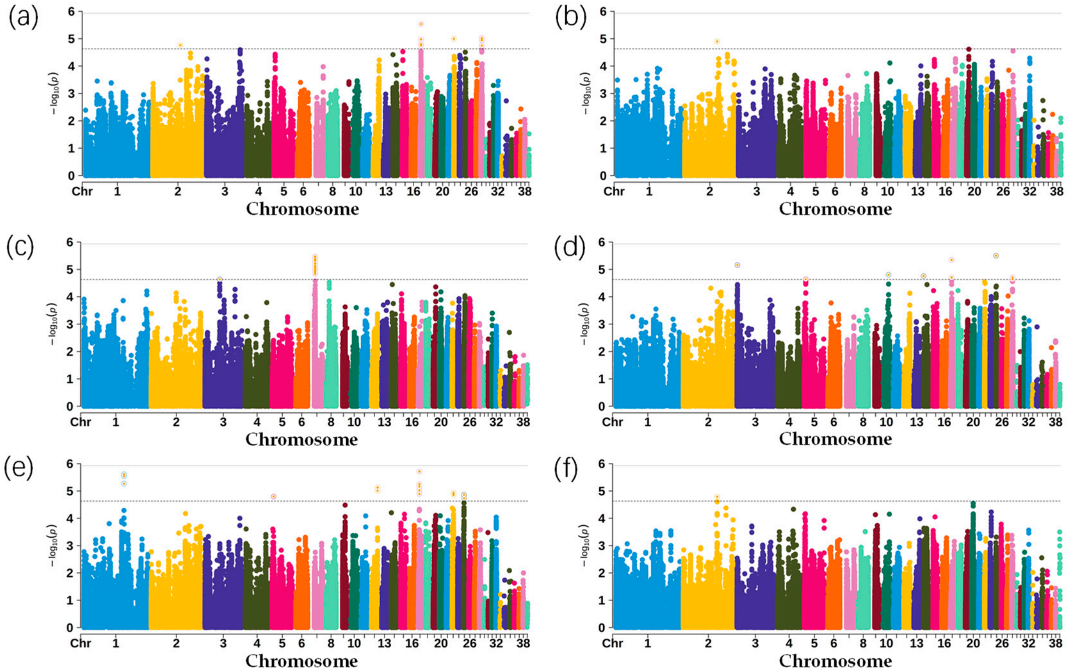

Figure S3. Manhattan plot of genome wide association study for egg production traits. Note: (a): EN1, egg number in the pre-peak laying period from 22 to 30 weeks of age; (b): EN2, egg number in the peak laying period from 31 to 55 weeks of age; (c): EN3, egg number in the persistent laying period from 56 to 66 weeks of age; (d): EN22-36WK, egg number from 22 to 36 weeks of age; (e): EN22-51WK, egg number from 22 to 51 weeks of age; (f): LR, egg laying rate from 28 to 56 weeks of age. Each dot represents a SNP in the dataset. The horizontal gray line and gray dashed line indicate the genome-wise significance threshold (P-value =1.17×10^{-6}) and genome-wise suggestive significance threshold (Pvalue = 2.35×10^{-5}), respectively.

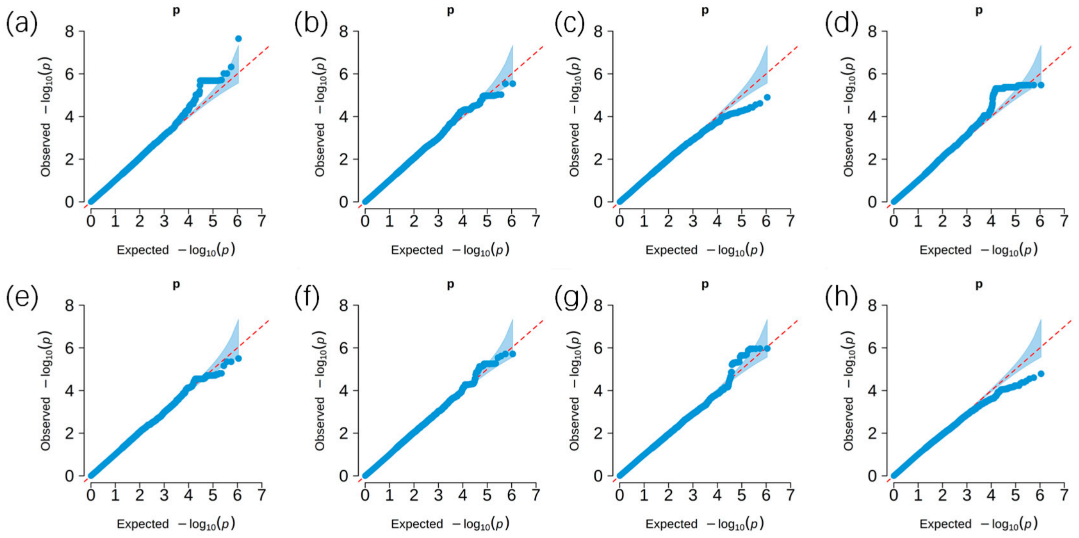

Figure S4. QQ plot of genome wide association study for egg production traits. Note: In this study, based on the results of association analysis for 8 traits, the range of the genomic inflation factor (Lambda) varied from 0.970 to 1.013. (a): AFE, age at first egg; (b): EN1, egg number in the pre-peak laying period from 22 to 30 weeks of age; (c): EN2, egg number in the peak laying period from 31 to 55 weeks of age; (d): EN3, egg number in the persistent laying period from 56 to 66 weeks of age; (e): EN22-36WK, egg number from 22 to 36 weeks of age; (f): EN22-51WK, egg number from 22 to 51 weeks of age; (g): EN22-66WK, egg number from 22 to 66 weeks of age; (h): LR, egg laying rate from 28 to 56 weeks of age. Each dot represents a SNP in the dataset.

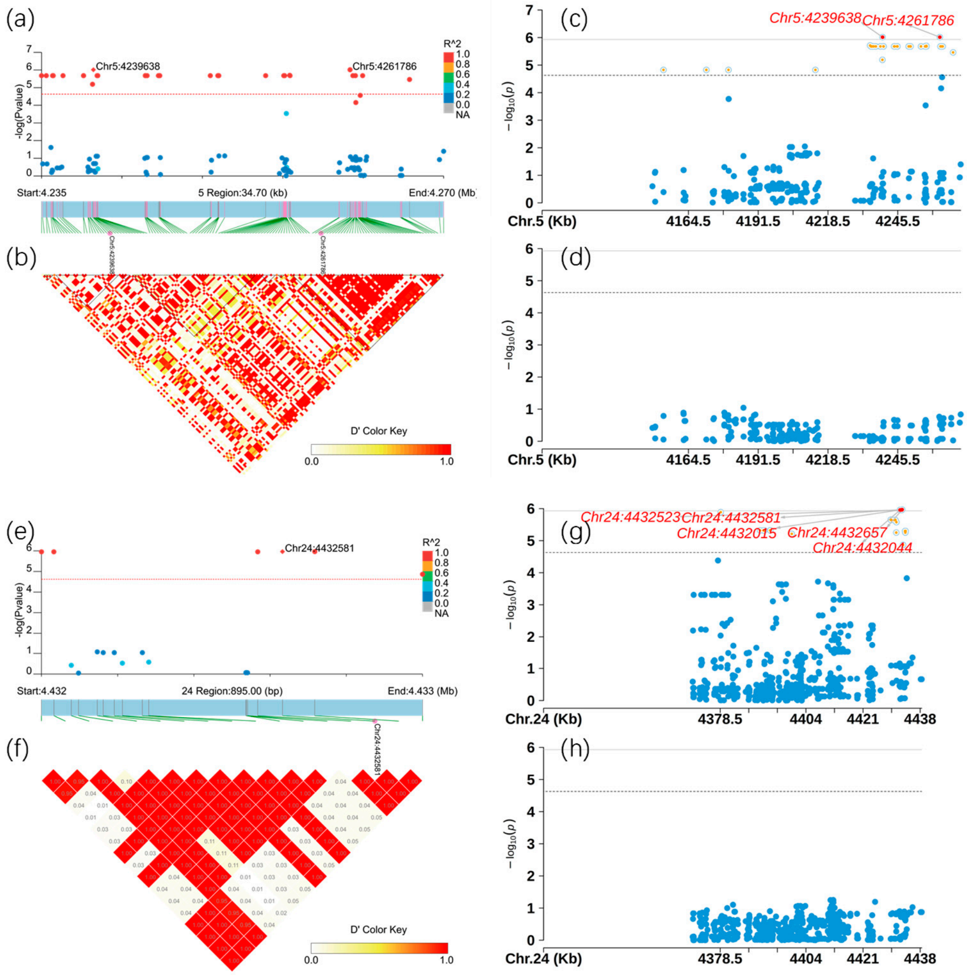

Figure S5. Manhattan plot of association analysis for age at first egg and egg number from 22 to 66 weeks of age in specific intervals, linkage disequilibrium analysis, and conditional analysis. The analysis of association and linkage disequilibrium (LD) between the interval 4.235Mb-4.270Mb on chromosome 5 and the age at first egg. On chromosome 5, the R^2 value revealed the degree of linkage disequilibrium between two loci, with the most significant SNP site (Chr:5:4239638, P-value=9.68 × 10^{-7}) shown as a red square in figure (a). The linkage disequilibrium measure D' for the same region is shown in figure (b). The Manhattan plots before and after the conditional analysis for the 4.15Mb-4.27Mb interval on chromosome 5 are presented in figures (c) and (d) respectively. The analysis of association and linkage disequilibrium for the interval 4.432Mb-4.433Mb on chromosome 24 with egg production between 22-66 weeks of age. On chromosome 24, the R^2 value revealed the degree of linkage disequilibrium between two loci, with the most significant SNP site (Chr:24:4432581, P-value=1.08 × 10^{-6}) highlighted as a red square in figure (e). Figure (f) displays the LD measure D' for that region. The Manhattan plots before and after the conditional analysis for the 4.37Mb-4.44Mb interval on chromosome 24 are shown in figures (g) and (h) respectively. The red dashed line represents the genome-wise suggestive significance threshold (P-value =2.35 × 10^{-5}). The horizontal gray line and gray dashed line indicate the genome-wise significance threshold (P-value =1.17 × 10-6) and genome-wise suggestive significance threshold (Pvalue = 2.35 × 10-5), respectively.
